# Transcriptomics-based drug repositioning in a neurocysticercosis rat model identifies putative therapeutic candidates reported to mitigate neuroinflammation and neurodegeneration

**DOI:** 10.21203/rs.3.rs-11408185/v1

**Published:** 2026-10-21

**Authors:** Omar Melo, Oksana Huerta, Karla Villalobos, Nancy Chile, Sneider A. Gutierrez Guarnizo, Hugo Naya, Cesar M. Gavidia, Hector H. Garcia, Robert H. Gilman, Manuela Verastegui

## Abstract

Neurocysticercosis (NCC) is a major cause of epilepsy and neurological symptoms in low- and middle-income countries. Although antiparasitic treatment targets the parasite, it often fails to fully resolve symptoms and can exacerbate brain tissue damage. Using a rat model of NCC and transcriptomic profiling, we assessed the effects of infection and treatment on brain gene expression to uncover disease molecular pathways and identify putative therapeutic candidates through in silico drug repurposing. We analyzed transcriptomic profiles at two stages of infection (4- and 6-month post-infection (MPI)), and after treatment at 6 MPI (6 MPI + T). At 6 MPI, fewer immune-related genes and pathways were enriched compared to 4 MPI, likely due to MAPK pathway repression, but immune activation increased significantly after treatment, possibly due to cyst degeneration. Drug repositioning identified 30 candidate drugs. Among these, vorinostat, ciclosporin, papaverine, valproic acid, and chlorpromazine have been reported to mitigate neuroinflammation and neurodegeneration. Taken together, these results provide a molecular rationale for drug repositioning in neurocysticercosis; however, further experimental validation in larger cohorts is required to confirm the therapeutic potential of the identified candidates.

